# Effects of neostigmine on postoperative neurocognitive dysfunction: a systematic review and meta-analysis

**DOI:** 10.3389/fnins.2025.1464272

**Published:** 2025-03-07

**Authors:** Xuelei Zhou, Linlin Chen, Li Zhao, Wei Mao, Xianchun Liu, Longyi Zhang, Ying Xie, Linji Li

**Affiliations:** Department of Anesthesiology, The Second Clinical Medical College, North Sichuan Medical College, Beijing Anzhen Nanchong Hospital, Capital Medical University & Nanchong Central Hospital, Nanchong, China

**Keywords:** neostigmine, acetylcholinesterase inhibitor, PND, dNCR, POD, POVN

## Abstract

**Introduction:**

Postoperative neurocognitive dysfunction (PND) is a common and serious complication following surgery. Neostigmine, an acetylcholinesterase inhibitor commonly administered during anesthesia to reverse residual neuromuscular blockade, has been suggested in recent studies to potentially reduce the incidence of PND. However, findings have been inconsistent across studies. Therefore, this study conducts a systematic review and meta-analysis to evaluate the effect of neostigmine on PND.

**Methods:**

We conducted a comprehensive literature search across multiple databases, including PubMed, EmBase, Web of Science, Cochrane Library, Scopus, SinoMed, and CNKI, to identify all relevant studies for inclusion. We included randomized controlled trials and cohort studies in our analysis. The risk of bias was assessed using the Risk of Bias 2 tool for randomized trials and the ROBINS-I tool for cohort studies.

**Results:**

A total of 11 studies were included in this analysis, consisting of 8 randomized controlled trials and 3 cohort studies. The incidence of PND was significantly lower in the neostigmine group compared to the control group (log(OR): −0.54, 95% CI [−1.04, −0. 05]; OR: 0.58, 95% CI: [0.35, 0.95], *p* = 0.03, I^2^ = 81.95%). Sensitivity analysis led to the exclusion of one cohort study. Consequently, the final meta-analysis comprised 10 studies, encompassing a total of 50,881 participants. The results indicate that the incidence of PND was significantly lower in the neostigmine group compared to the control group (log(OR):−0. 27, 95% CI [−0.47, −0. 08]; OR: 0.76, 95% CI: [0.62, 0.91], *p* = 0.01, I^2^ = 2.50%). However, Meta-analysis of RCTs and cohort studies showed no significant difference. Subgroup analysis indicated that neostigmine reduced the incidence of delayed neurocognitive recovery (dNCR), but its impact on POD was unclear, with no significant association to nausea and vomiting. These findings suggest that neostigmine may reduce the risk of PND, but caution is needed in interpretation.

**Conclusion:**

Neostigmine may have a potential positive effect in reducing the incidence of PND. However, no statistical difference was observed when meta-analyses were performed separately for randomized controlled trials (RCTs) and cohort studies. Given the limited number of studies available and the limitations of the current research, further investigation is needed to clarify the impact of neostigmine on PND.

**Systematic review registration:**

https://www.crd.york.ac.uk/PROSPERO/view/CRD42024537647, Identifier CRD42024537647.

## 1 Introduction

Globally, approximately 310 million surgeries are performed annually to address the needs of patients with surgical diseases (Rose et al., 2015), and this demand continues to rise (Perera et al., 2021). However, postoperative complications affect approximately 16 million individuals each year, posing a significant threat to patient health and substantially increasing the burden on healthcare systems (Dobson, 2020). One of the most common postoperative complications is postoperative neurocognitive dysfunction (PND), which includes postoperative delirium (POD), delayed neurocognitive recovery (dNCR), and postoperative neurocognitive disorder (Evered et al., 2018). The incidence of PND ranges from 11 to 51% (Inouye et al., 2014; Mahanna-Gabrielli et al., 2019). Postoperative neurocognitive dysfunction is associated with a higher occurrence of postoperative complications, increased mortality, decreased quality of life, prolonged hospital stays, and increased healthcare costs (Inouye et al., 2014; Pandharipande et al., 2017; Wilson et al., 2020). POD is defined as a transient neuropsychiatric syndrome that occurs within 7 days after surgery, characterized by fluctuating disturbances in cognition, consciousness, and attention (Evered et al., 2018). In contrast, dNCR refers to the emergence of new cognitive impairments within 30 days after complete recovery of consciousness postoperatively (Evered et al., 2018).

Recent studies indicate that implementing appropriate perioperative measures to prevent PND is vital for improving patient outcomes (Strøm et al., 2014; Liu et al., 2022; Swarbrick and Partridge, 2022). Multiple studies have indicated that a decrease in acetylcholine levels and an increase in cholinesterase activity are closely associated with the onset of PND, suggesting a potential underlying pathological mechanism (Adam et al., 2020; Downes and Granato, 2004; Cerejeira et al., 2011; John et al., 2017; Cerejeira et al., 2012; Cheng et al., 2022). Acetylcholinesterase breaks down acetylcholine in the synaptic cleft, playing an indispensable role in neurotransmission. If its function is compromised, neurotransmission may be disrupted, potentially leading to PND (Downes and Granato, 2004). Previous research has found that anticholinergic drugs can induce delirium by antagonizing cholinergic neurotransmission (Adam et al., 2020). Recent studies have also discovered that cholinesterase inhibitors can prevent PND by mitigating inflammatory responses and oxidative stress (Deng et al., 2019; Zhang et al., 2019; Umholtz and Nader, 2017).

Neostigmine, an acetylcholinesterase inhibitor, is commonly used during anesthesia to reverse residual neuromuscular blockade. While previously thought unable to penetrate the blood–brain barrier, recent research suggests that surgical procedures and anesthesia can increase blood–brain barrier permeability through the induction of systemic inflammation and stress responses (Saxena and Maze, 2018). This phenomenon may facilitate the passage of these compounds (Taylor et al., 2022). Hence, acetylcholinesterase inhibitors may serve as potential agents for preventing PND (Swarbrick and Partridge, 2022). However, current clinical studies present conflicting findings. This study aims to evaluate, through the integration of existing literature data, the impact of postoperative neostigmine administration on the incidence of PND compared to placebo, sugammadex, and natural drug metabolism in control groups. The outcomes of this research are anticipated to offer crucial insights for the clinical management of PND. Moreover, this study aims to provide new perspectives on the role of neostigmine in PND, potentially influencing overall treatment outcomes and enhancing the quality of life for surgical patients.

## 2 Methods

### 2.1 Study design

This study rigorously follows the guidelines of the Preferred Reporting Items for Systematic Reviews and Meta-Analyses (PRISMA) statement (Page et al., 2021). As our data are exclusively derived from published literature, ethical review is not applicable. Additionally, our study is registered in the international prospective register of systematic reviews (PROSPERO) under registration identifier CRD42024537647.

### 2.2 Literature search

XZ conducted a systematic search of PubMed, Embase, Web of Science, Cochrane Library, Scopus, SinoMed, and CNKI to comprehensively include relevant literature. Our search strategy utilized a combination of free-text and MeSH terms, encompassing perioperative neurocognitive disorders, postoperative delirium, cognitive function, delayed neurocognitive recovery, postoperative cognitive dysfunction, and neostigmine (Supplementary Figure 1 for detailed search strategy).

### 2.3 Study selection

Two researchers LC and WM, independently assessed and reviewed the titles, abstracts, and full texts of the papers to select those that met the inclusion criteria. Any discrepancies that arose during this process were resolved through discussion. If a consensus could not be reached, a third researcher, LZ was involved in the decision-making. Given the limited number of randomized controlled trials (RCTs) on this topic, and the evidence indicating that well-designed cohort studies are comparable to RCTs in assessing treatment effects (Concato et al., 2000; Golder et al., 2011; Schwingshackl et al., 2021), cohort studies were also included in this research. The inclusion criteria for the studies were as follows:

Patients undergoing surgery.

The intervention group will receive neostigmine treatment.

The control group includes patients receiving sugammadex, those using a placebo (such as saline), and patients awaiting the natural metabolism of neuromuscular blocking agents (NMBAs).

Assessments will be conducted for postoperative neurocognitive dysfunction.

The exclusion criteria for the studies were as follows:

Literature classified as case reports.

Review articles.

Trial protocols.

Literature with insufficient or unclear data.

Full texts that were inaccessible or where the authors could not be contacted.

### 2.4 Data extraction and integration

We initially created a data extraction form and conducted a pilot test to refine it. Subsequently, two independent researchers performed the data extraction, and any discrepancies were discussed. If the two researchers could not reach a consensus, a third researcher made the final decision. The data extraction form included the following information: author, publication year, study design, participants’ age, number of participants, type of surgery, neuromuscular blocking agent, neostigmine usage and dosage, and the incidence of nausea and vomiting (POVN). We employed WebPlotDigitizer (version 5; WebPlotDigitizer, A. Rohatgi, Pacifica, CA, USA) to extract data from graphical representations, only after unsuccessful attempts to contact the original study authors for additional data. To estimate the mean and standard deviation for data described by the median and interquartile range, we applied the equations provided by Wan et al. (2014).

### 2.5 Bias risk assessment

We used the Cochrane Collaboration’s Risk of Bias 2 tool (Sterne et al., 2019), to assess RCTs for random sequence generation, allocation concealment, blinding of participants, blinding of healthcare providers, blinding of data collectors, blinding of outcome assessors, incomplete outcome data, selective outcome reporting, and other sources of bias. For cohort studies, we used the ROBINS-I tool to evaluate the risk of bias and visualized the results using the platform available at https://mcguinlu.shinyapps.io/robvis (Sterne et al., 2016; McGuinness and Higgins, 2020). Bias risk assessment was conducted independently by researchers LC and XZ, with any discrepancies discussed until consensus was achieved. This process will ensure the reliability and accuracy of the assessment results.

### 2.6 Data analysis methods

We utilized Stata 17.0 and Review Manager 5.4 software for data analysis. To evaluate heterogeneity among the studies, we employed τ^2^ (Tau squared) and I^2^ (I-squared) statistics. These measures help quantify the level of heterogeneity in the data, facilitating a more accurate interpretation of the results. To minimize the impact of confounding factors and better reflect real-world conditions, we adopted a random-effects model (Borenstein et al., 2010). Moreover, in cases of very low heterogeneity, the random-effects model produces results similar to those of the fixed-effects model (Borenstein et al., 2010). Therefore, our analysis consistently applied the random-effects model to calculate and aggregate the log odds ratios [log(OR)] and their 95% confidence intervals (CI) for binary outcomes. Lastly, we used funnel plots and Egger’s test to assess and detect publication bias for each evaluated outcome (Cumpston et al., 2019).

## 3 Results

### 3.1 Inclusion of studies

**Figure 1:**
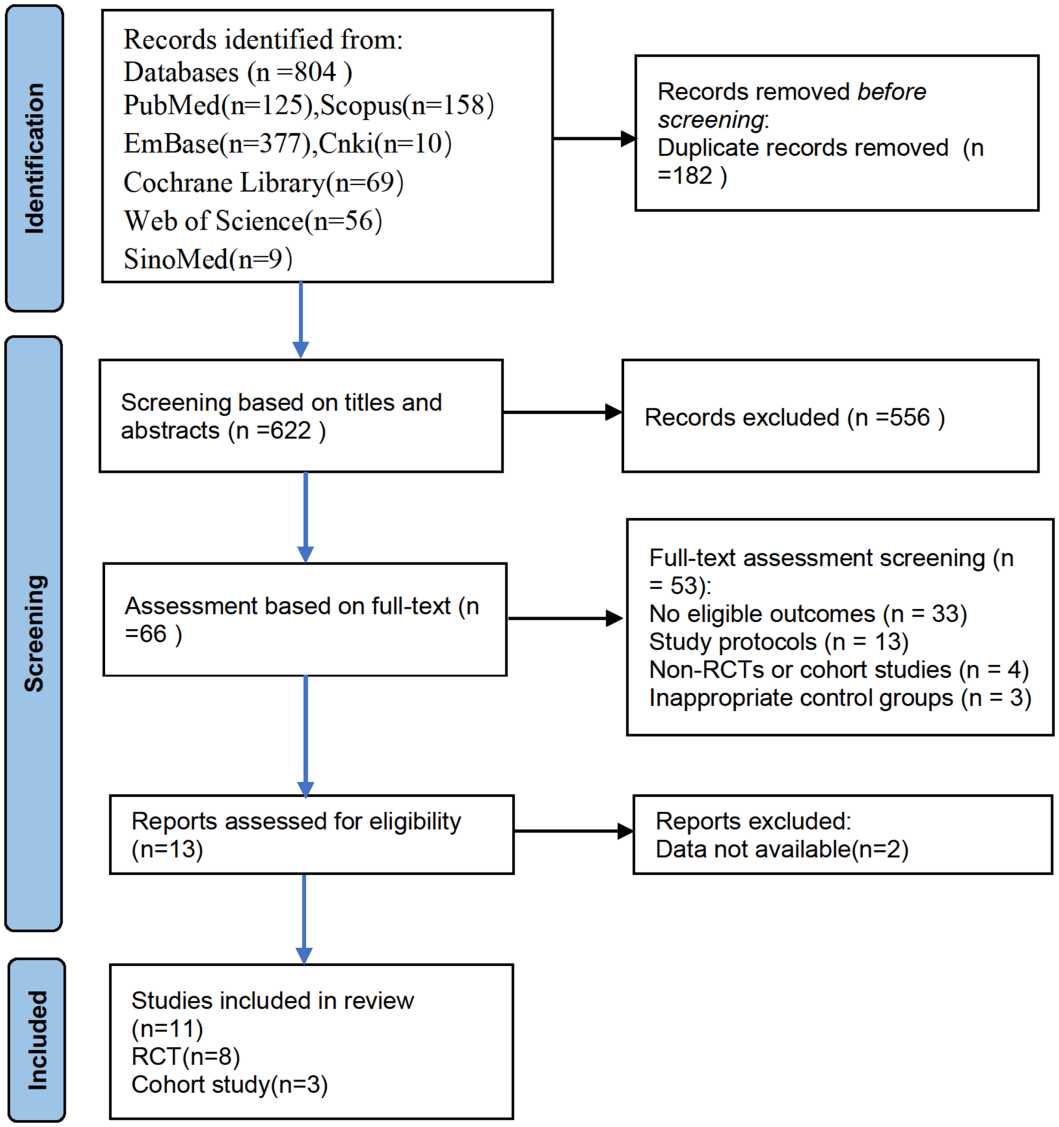
Flow diagram of study selection.

Researchers conducted searches in the following databases: PubMed (*n* = 125), EMBASE (*n* = 377), Cochrane Library (*n* = 69), Web of Science (*n* = 56), Scopus (*n* = 158), SinoMed (*n* = 9), and CNKI (*n* = 10), resulting in a total of 804 articles. We excluded 182 duplicate articles. During the study selection process, the two researchers initially screened 622 articles based on their titles and abstracts, excluding 556. Subsequently, a full-text review of the remaining 66 articles was conducted, with 53 articles being excluded. It was noted that two articles, although assessing cognitive function, did not provide the incidence of PND, and were therefore excluded (Cao et al., 2023; Piskin et al., 2016). After rigorous screening, 11 articles were ultimately included, consisting of 8 RCTs and 3 cohort studies (Figure 1). During the literature review, we identified two studies that conducted neurocognitive function assessments but did not report the incidence of dNCR. Attempts to contact the authors for further data were unfortunately unsuccessful, leading to the exclusion of these studies from our analysis. Additionally, both studies compared the effects of neostigmine and sugammadex on dNCR and found no statistically significant difference between the two (Cao et al., 2023; Piskin et al., 2016).

### 3.2 Study characteristics

The detailed characteristics of the included studies are presented in Table 1. These studies were published between 2015 and 2024. Four studies compared the effects of neostigmine with a placebo (Deng et al., 2024; Purohit et al., 2022; Zhu et al., 2020; Shuai and Guan Yun, 2015); four studies compared neostigmine with sugammadex (Batistaki et al., 2017; Brueckmann et al., 2015; Rössler et al., 2024; Oh et al., 2016); and three studies included control groups that received no treatment (Hang et al., 2023; Jing et al., 2016; Zhu, 2017). The types of surgeries varied: three studies focused on laparoscopic surgeries; two on hip fracture and hip replacement surgeries; two on non-cardiac surgeries; two on gastrointestinal tumor surgeries; one study excluded patients undergoing neurosurgery, cardiac, vascular, or orthopedic surgeries; and one study did not specify the type of surgery.

**Table 1:** The characteristics of included studies.

Source	Study design	Surgery	NMBAs	Postoperative diagnostic criteria and timing	Experimental				Control			
					Age (years)	Sample size	Drugs	Dosage	Age (years)	Sample size	Drugs	Dosage
Deng et al. (2024)	RCT	Noncardiac surgery	Cisatracurium	CAM, MMSE and MoCA; Day 1	71 ± 6.09	56	Neostigmine	0.04 mg/kg	71.3 ± 6.84	56	NS	/
Purohit et al. (2022)	RCT	Colon carcinoma surgery	Cisatracurium	CAM, MDAS;/	64.19 ± 10.51	196	Neostigmine	0.04 mg/kg	63.34 ± 10.38	205	Placebo	/
Zhu et al. (2020)	RCT	Radical section of gastrointestinal cancer	Cisatracurium	MMSE; Day 7	72.9 ± 6.1	78	Neostigmine	0.04/0.02 mg/kg	72.9 ± 4.9	42	NS	/
Batistaki et al. (2017)	RCT	Non-neurosurgical, cardiac, vascular, or orthopedic surgery	Rocuronium	MMSE; At Discharge	61.25 ± 1.09	82	Neostigmine	0.04 mg/kg	61.64 ± 1.37	78	Sugammadex	2 mg/kg
Brueckmann et al. (2015)	RCT	Laparoscopic or open abdominal surgery	Rocuronium	/; /	57.0 ± 12.7	77	Neostigmine	0.02–0.08 mg/kg	56.4 ± 12.8	74	Sugammadex	2–4 mg/kg
Hang et al. (2023)	RCT	Laparoscopic surgery	Cisatracurium	MMSE, MoCA; Day 1	70.2 ± 10.2	71	Neostigmine	0.04 mg/kg	75.2 ± 5.1	50	No treatment	/
Jing et al. (2016)	RCT	Laparoscopic cholecystectomy	/	MMSE; Days 1 and 3	53.8 ± 2.71	44	Neostigmine	2 mg	54.22 ± 1.71	22	No treatment	/
Shuai and Guan Yun (2015)	RCT	/	Cisatracurium	/; /	17.56 ± 10.40	18	Neostigmine	0.02 mg/kg	18.14 ± 10.25	18	NS	/
Rössler et al. (2024)	Cohort study	Noncardiac surgery	/	CAM; Days 1 and 4	57.6 ± 15.4	42,578	Neostigmine	/	59.8 ± 15.3	6,881	Sugammadex	/
Zhu (2017)	Cohort study	Hip replacement	Cisatracurium	MMSE; Day 7	73.5 ± 8.5	775	Neostigmine	1–2 mg	74.5 ± 7.5	597	No treatment	/
Oh et al. (2016)	Cohort study	Hip fracture surgery	Rocuronium	CAM; At Discharge	75 ± 9	96	Neostigmine	0.05 mg/kg	76 ± 7	78	Sugammadex	2 mg/kg

Age is presented as mean ± standard deviation; RCT, Randomized controlled study; NMBAs, Neuromuscular blocking agents; CAM, Confusion Assessment Method; MOCA, Montreal Cognitive Assessment; MDAS, Memorial Delirium Assessment Scale; MMSE, Mini-Mental State Examination; NS, Normal saline; /no available data.

Various methods were employed to diagnose PND in the included studies, including the Confusion Assessment Method (CAM), Mini-Mental State Examination (MMSE), Montreal Cognitive Assessment (MOCA), and Memorial Delirium Assessment Scale (MDAS) (Wong et al., 2010; Adamis et al., 2010). In the studies we reviewed, seven articles reported the overall incidence of PND (Purohit et al., 2022; Zhu et al., 2020; Shuai and Guan Yun, 2015; Batistaki et al., 2017; Brueckmann et al., 2015; Oh et al., 2016; Zhu, 2017). Unfortunately, some of these studies did not specify the time of PND diagnosis (Purohit et al., 2022; Shuai and Guan Yun, 2015; Batistaki et al., 2017; Brueckmann et al., 2015). Four studies documented the incidence of PND on the first postoperative day (Deng et al., 2024; Rössler et al., 2024; Hang et al., 2023; Jing et al., 2016), while two studies reported PND incidence on the third postoperative day (Deng et al., 2024; Jing et al., 2016). Due to the variability in the timing of PND assessments across studies, we included data from studies that either reported the overall incidence of PND or the incidence on the first postoperative day for further analysis.

### 3.3 Risk of bias

**Figure 2:**
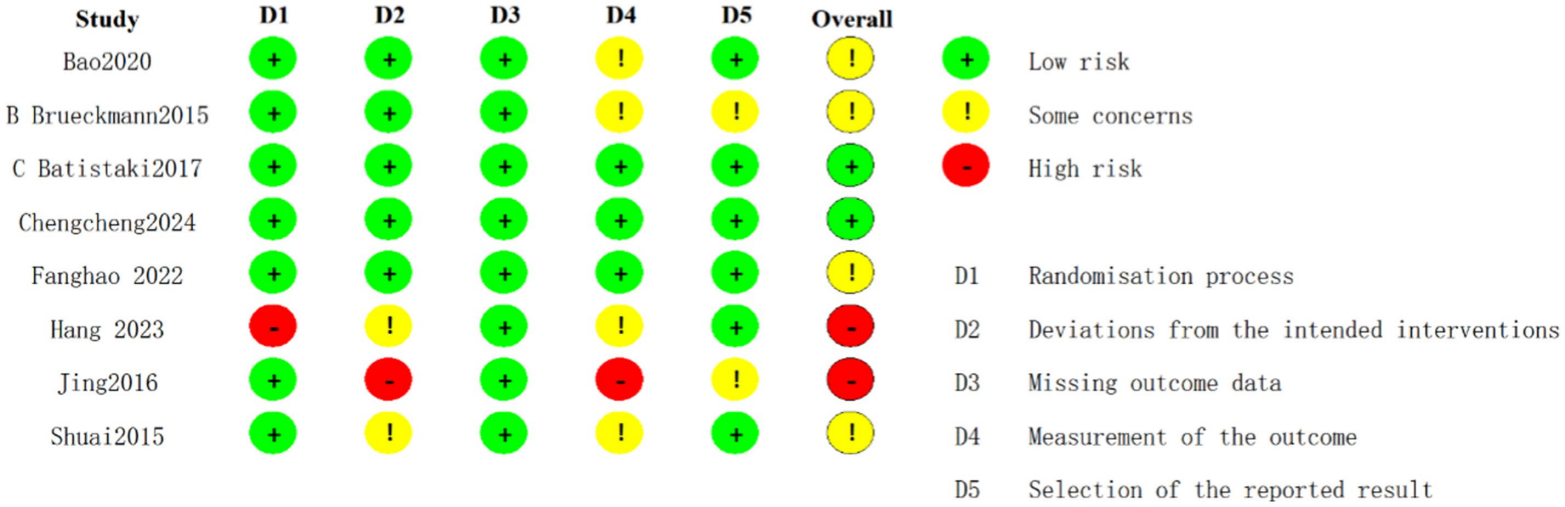
Risk of bias summary for RCTs.

**Figure 3:**
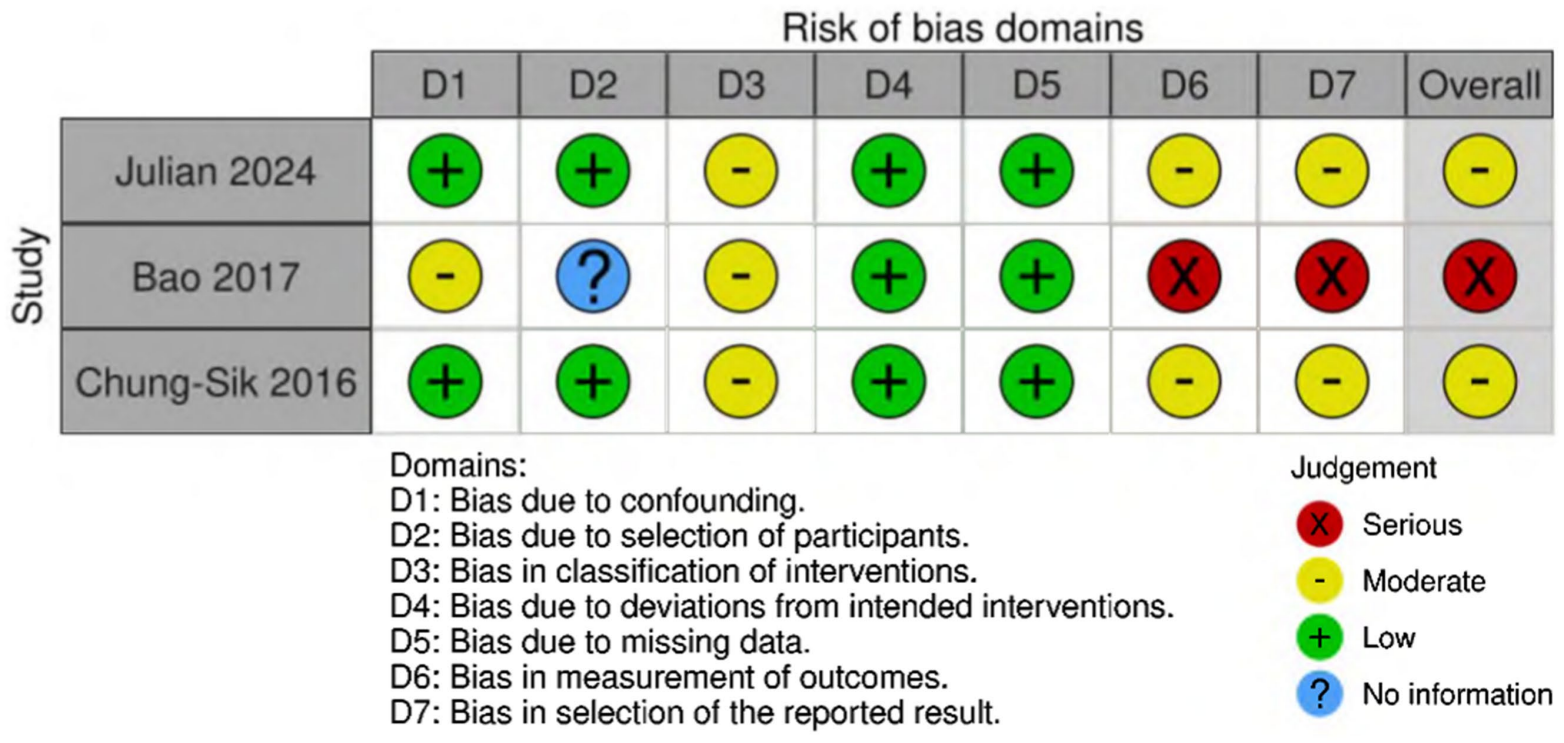
Risk of bias summary for cohort studies.

Among the randomized controlled trials included, two were assessed as having a low risk of bias (Deng et al., 2024; Batistaki et al., 2017), four were identified as having a moderate risk (Purohit et al., 2022; Zhu et al., 2020; Shuai and Guan Yun, 2015; Brueckmann et al., 2015), and two were judged to have a high risk of bias (Brueckmann et al., 2015; Hang et al., 2023) (Figure 2). The quality assessment of cohort studies shows that Rössler et al. (2024) and Oh et al. (2016) have a low to moderate risk of bias, while Zhu (2017) has a higher risk of bias and should be interpreted with caution (Figure 3).

#### 3.3.1 The impact of neostigmine on PND

**Figure 4:**
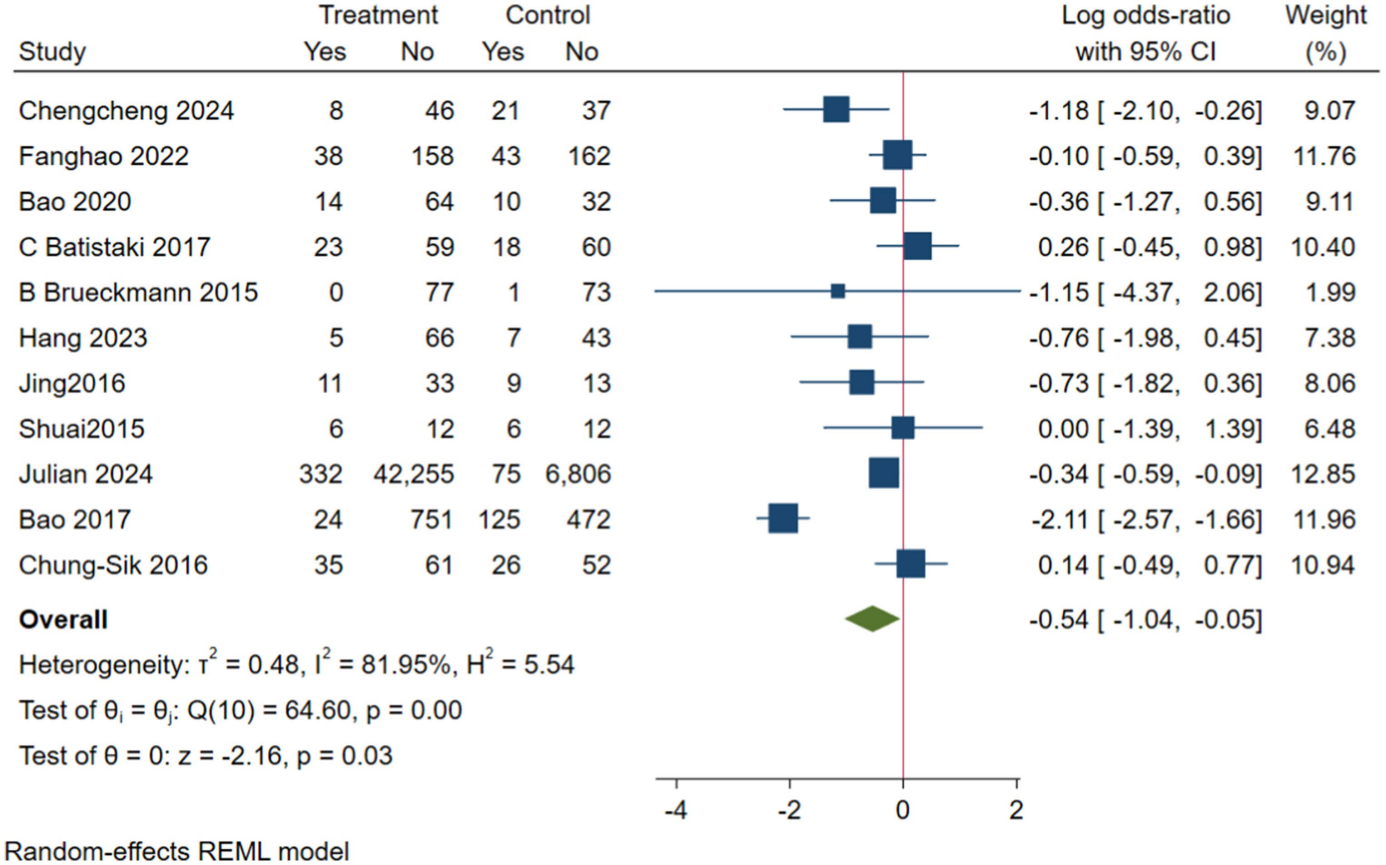
Forest map of the effects of neostigmine on PND.

**Figure 5:**
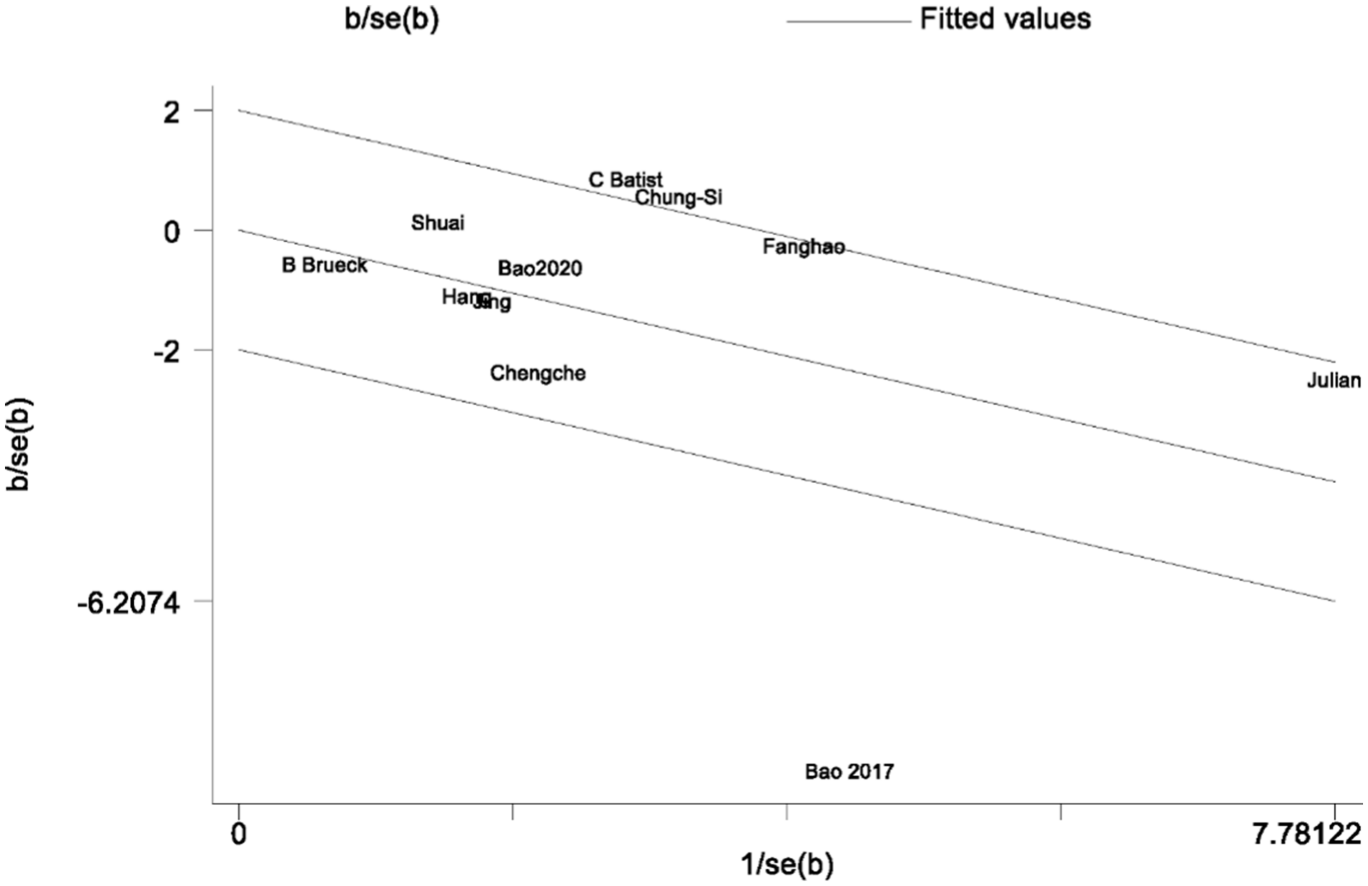
Galbraith plot of PND.

**Figure 6:**
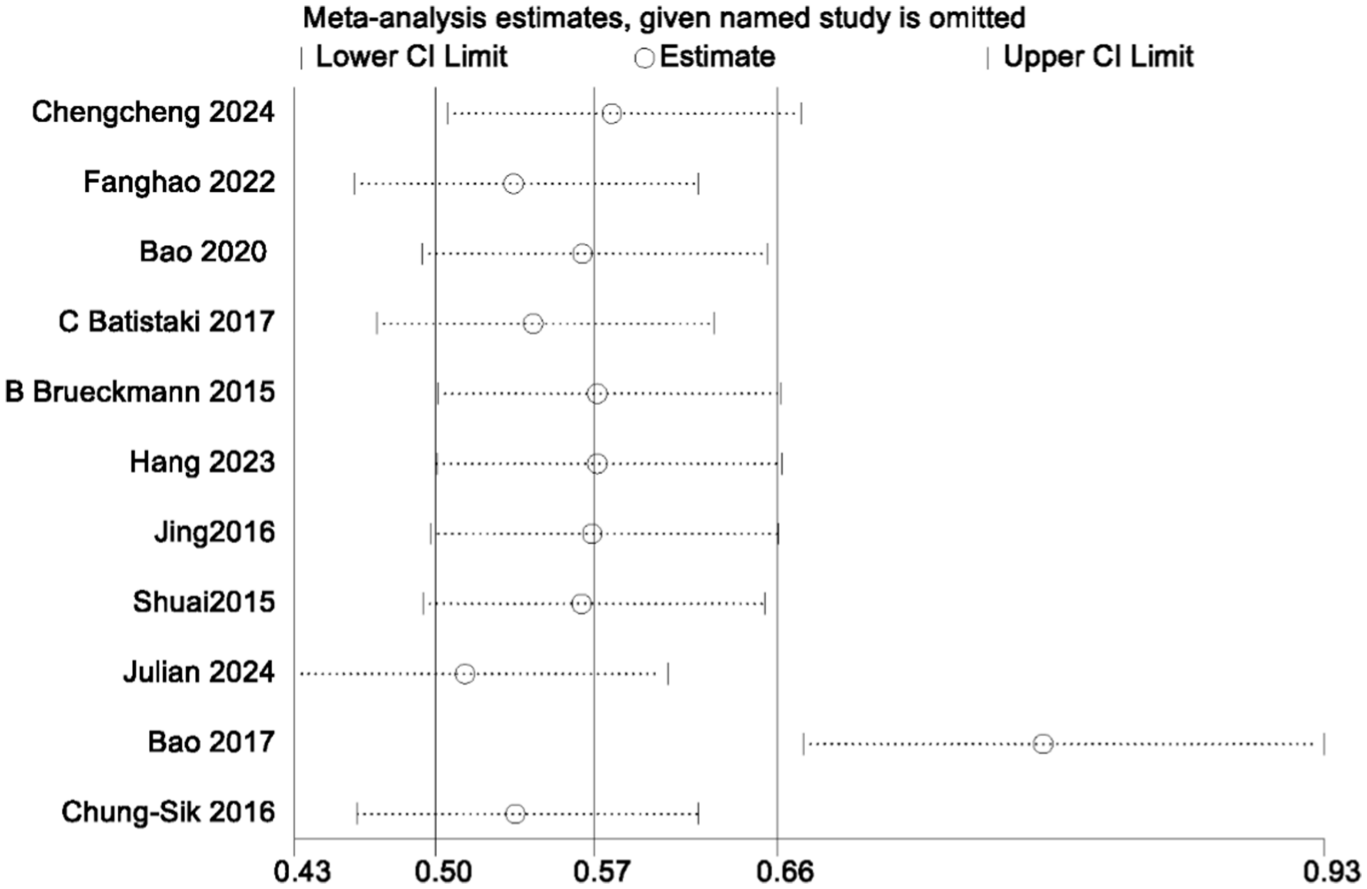
Plot of individual study effects of PND on results.

In the analysis of PND, a total of 11 studies were included, consisting of eight randomized controlled trials and three cohort studies. The findings demonstrated that the incidence of PND in the neostigmine group was significantly lower compared to the control group (log(OR): −0. 54, 95% CI [−1.04, −0. 05]; OR: 0.58, 95% CI: [0.35, 0.95], *p* = 0.03, *I*^2^ = 81.95%), as depicted in Figure 4. However, the *I*^2^ value of 81.95% indicates substantial heterogeneity among the included studies. To elucidate the sources of this heterogeneity, we employed a Galbraith plot (Figure 5) and performed an influence analysis of individual studies on the overall outcomes (Figure 6).

**Figure 7:**
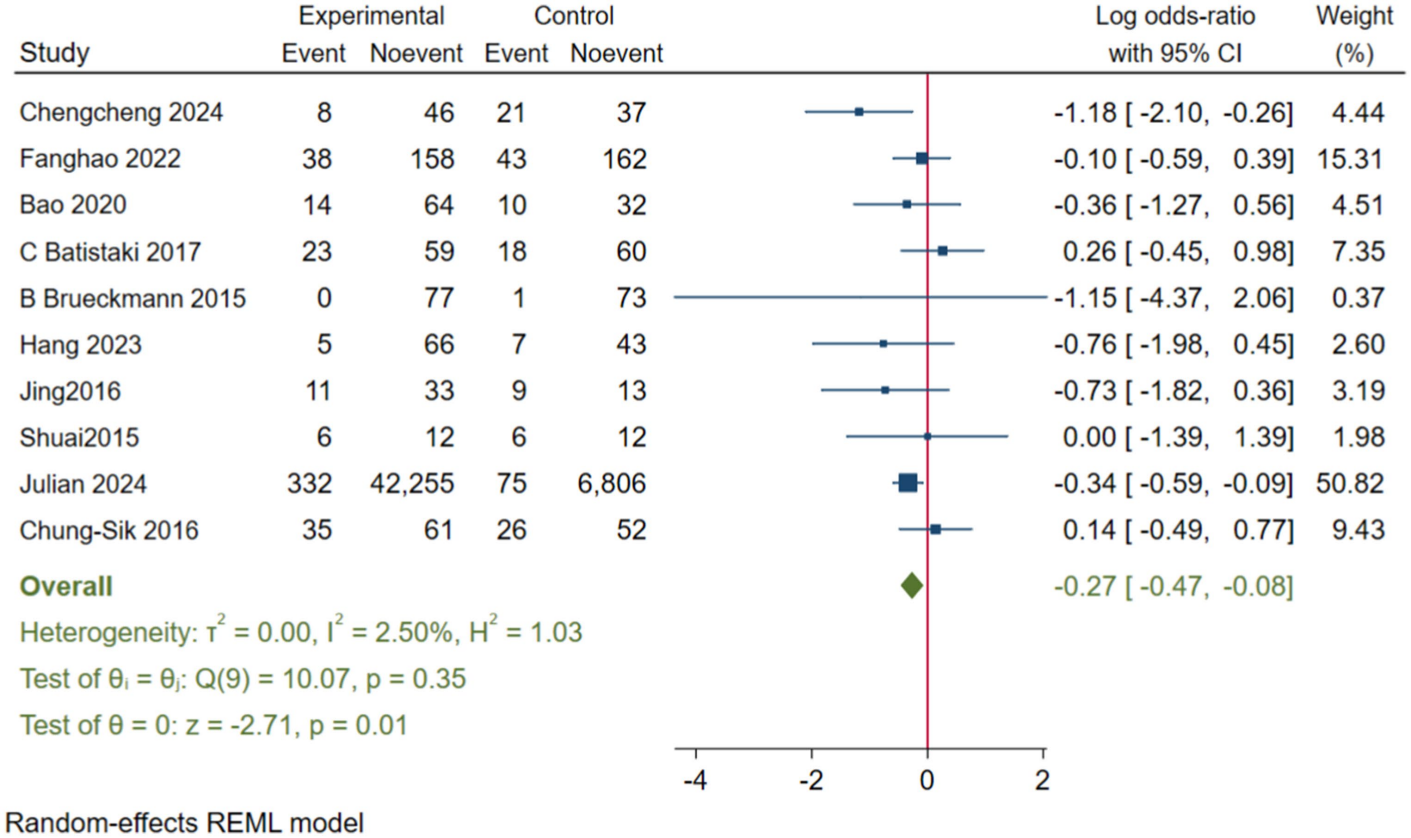
Forest map of the effect of neostigmine on PND after sensitivity analysis.

To enhance the robustness and external validity of our findings, a sensitivity analysis was conducted. Zhu (2017) was excluded due to significant deviation in Figures 5 and a confidence interval in Figure 6 that did not align with the overall effect estimate. The revised analysis incorporated 8 randomized controlled trials (Deng et al., 2024; Purohit et al., 2022; Zhu et al., 2020; Shuai and Guan Yun, 2015; Batistaki et al., 2017; Brueckmann et al., 2015; Hang et al., 2023; Jing et al., 2016) and 2 cohort studies (Rössler et al., 2024; Oh et al., 2016), encompassing a total of 50,881 participants. The results continued to demonstrate a significantly lower incidence of PND in the neostigmine group compared to the control group (log(OR): −0. 27, 95% CI [−0.47, −0. 08]; OR: 0.76, 95% CI: [0.62, 0.91], *p* = 0.01, *I*^2^ = 2.50%), as illustrated in (Figure 7). The markedly reduced heterogeneity (*I*^2^ = 2.50%) was further validated using a Galbraith plot (Supplementary Figure 2). Additionally, to evaluate the potential for publication bias, a funnel plot was generated (Supplementary Figure 3), and Egger’s test was conducted (*p* = 0.664) (Supplementary Figure 4). The results from these analyses did not indicate significant evidence of publication bias. This comprehensive analysis strongly suggests that neostigmine is associated with a reduced risk of PND.

We performed separate meta-analyses for the RCTs and cohort studies, which showed no significant difference in postoperative PND incidence between the neostigmine and control groups. In the RCTs (log(OR): −0. 34, 95% CI [−0.73, 0.56]; OR: 0.71, 95% CI [0.48, 1.75], *p* = 0.09, I^2^ = 26.7%) (Supplementary Figure 5) and in the cohort studies (log(OR): −0. 78, 95% CI [−0.21, 0.56]; OR: 0.46, 95% CI [0.81, 1.75], *p* = 0.25, I^2^ = 96.6%) (Supplementary Figure 6), no significant differences were observed in the incidence of postoperative PND between the neostigmine and control groups.

#### 3.3.2 Subgroup analysis

**Figure 8:**
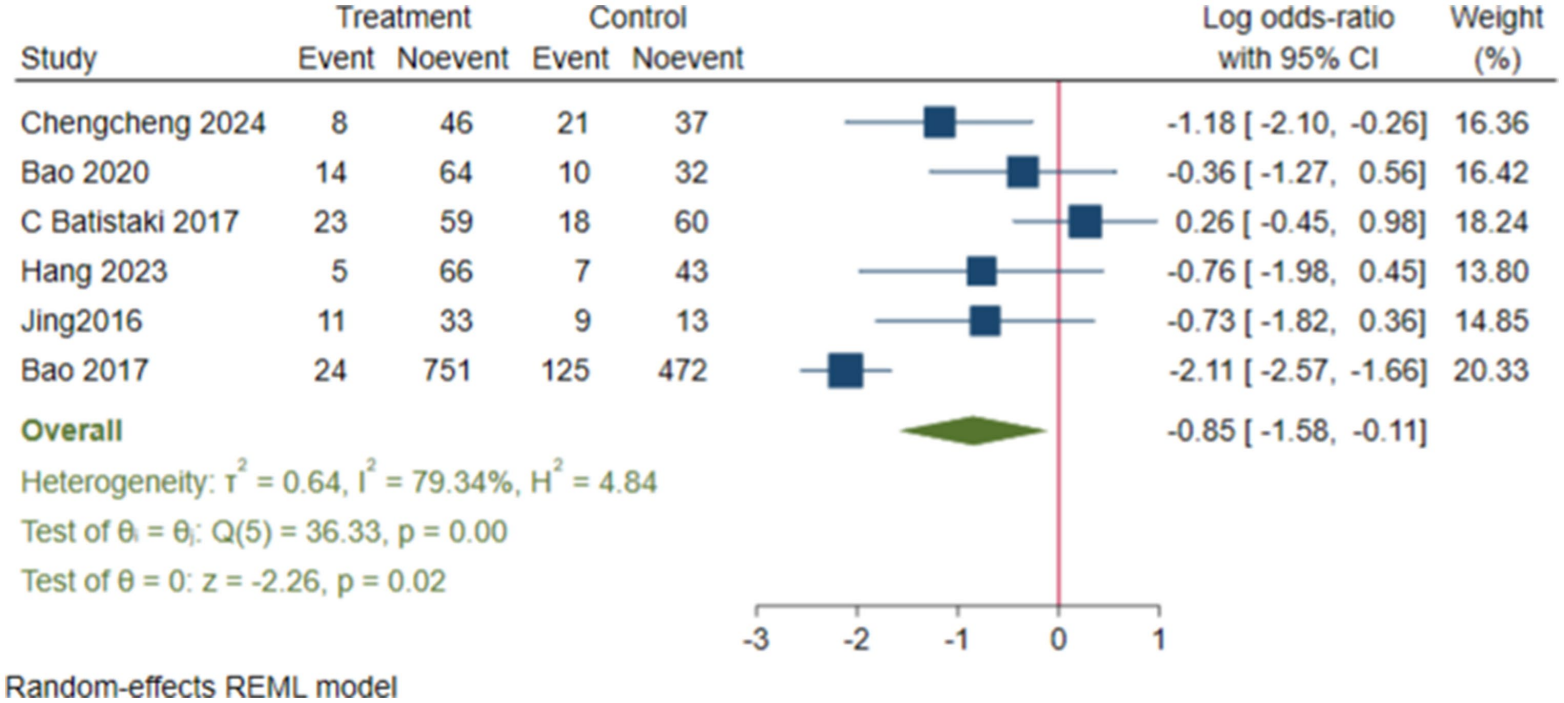
Forest map of the effects of neostigmine on dNCR.

We conducted subgroup analyses, and the results showed that the neostigmine group significantly reduced the incidence of dNCR compared to the control group (log(OR): −0. 85, 95% CI [−1.58, −0.11]; OR: 0.43, 95% CI: [0.21, 0.89], *p* = 0.02) (Figure 8) (Deng et al., 2024; Zhu et al., 2020; Batistaki et al., 2017; Hang et al., 2023; Jing et al., 2016; Zhu, 2017). In contrast, the incidence of POD showed a (log(OR) of −0. 21, 95% CI [−0.46, 0.03]; OR: 0.81, 95% CI: [0.63, 1.03], *p* = 0.09) (Purohit et al., 2022; Shuai and Guan Yun, 2015; Brueckmann et al., 2015; Rössler et al., 2024; Oh et al., 2016), compared with the control group (Supplementary Figure 7).

#### 3.3.3 The impact of neostigmine on POVN

**Figure 9:**
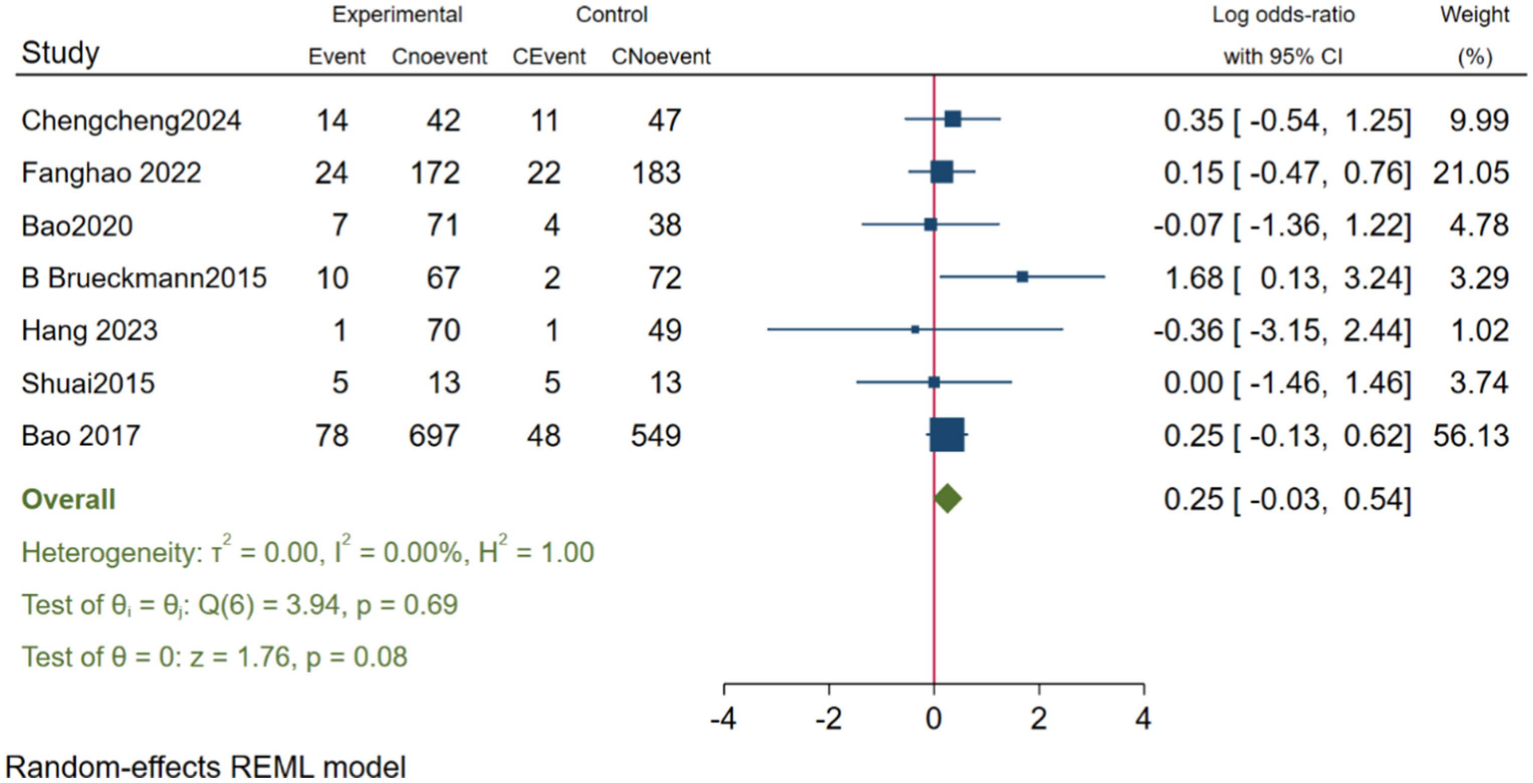
Forest map of effects of POVN.

In evaluating the impact of neostigmine on the incidence of PONV, we included seven studies Deng et al. (2024), Zhu (2017), Batistaki et al. (2017), Hang et al. (2023), Jing et al. (2016), and Zhu et al. (2020), encompassing a total of 2,315 participants. The analysis revealed that the incidence of PONV was slightly higher in the neostigmine group compared to the control group; however, this difference was not statistically significant (log(OR): 0.25, 95% CI [−0.03, 0.54];OR: 1.28, 95% CI: [0.97, 1.72], *p* = 0.08, *I*^2^ = 0.00%), as illustrated in Figure 9. Thus, while the incidence of PONV was higher in the neostigmine group, the difference did not reach statistical significance. Heterogeneity test Galbraith plot (Supplementary Table 8). Furthermore, publication bias was assessed using a funnel plot (Supplementary Table 9) and Egger’s test (*p* = 0.768) (Supplementary Table 10), which did not reveal any significant evidence of publication bias. However, due to the limitations of the included studies, there is a need for more large-scale, multicenter randomized controlled trials to further elucidate the relationship between neostigmine and the incidence of POVN.

## 4 Discussion

This meta-analysis suggests that neostigmine may have a positive effect in reducing the incidence of PND. However, no significant effect of neostigmine on PND was found when RCTs and cohort studies were analyzed separately. Subgroup analysis further revealed that neostigmine effectively reduced the incidence of dNCR. However, its effects on POD and PONV remain inconclusive, warranting further investigation. Additionally, there is a degree of heterogeneity in the included studies, encompassing variations in disease types, surgical procedures, patient ages, anesthesia protocols, and outcome measures. Consequently, the interpretation of these results should be approached with caution to ensure accuracy and reliability. Further high-quality, large-scale randomized controlled trials are necessary to confirm these findings and to better understand the potential benefits and risks associated with neostigmine use in the perioperative setting.

The initial meta-analysis included 11 studies: 8 RCTs and 3 cohort studies. The results indicated that the incidence of PND was significantly lower in the neostigmine group compared to the control group (log(OR): −0. 54, 95% CI [−1.04, −0. 05]; OR: 0.58, 95% CI: [0.35, 0.95], *p* = 0.03, *I*^2^ = 81.95%). The high I^2^ value of 81.95% suggests substantial heterogeneity, which could potentially bias the study results. Zhu (2017) were excluded because the results in Figure 4 significantly deviated from the pooled effect estimate, and the confidence intervals in Figure 5 were not consistent with the overall effect. In our sensitivity analysis, after excluding this study, we found that the overall results remained largely unchanged, but heterogeneity was significantly reduced. This suggests that the inclusion of Zhu (2017) may have introduced additional variability into the analysis. We also discussed potential differences between this study and other findings, particularly in outcome measurement: the study did not perform preoperative neurocognitive assessments, but instead, 1 week postoperatively, the surgeons observed signs of cognitive impairment in patients before administering the MMSE. This approach may have introduced bias. By excluding this study, our refined analysis included 8 RCTs (Deng et al., 2024; Purohit et al., 2022; Zhu et al., 2020; Shuai and Guan Yun, 2015; Batistaki et al., 2017; Brueckmann et al., 2015; Hang et al., 2023; Jing et al., 2016) and 2 cohort studies (Rössler et al., 2024; Oh et al., 2016). The adjusted meta-analysis results (log(OR): −0. 27, 95% CI [−0.47, −0.08]; OR: 0.76, 95% CI: [0.62, 0.91], *p* = 0.01, I^2^ = 2.50%) demonstrate that postoperative use of neostigmine may reduce the incidence of PND.

Neither the RCTs nor the cohort studies showed a significant association between neostigmine and a reduction in postoperative PND incidence in their respective meta-analyses. This may be attributed to factors such as the heterogeneity of study designs, insufficient sample size, the multifactorial nature of postoperative PND, and the potentially limited effect of neostigmine on PND. Our subgroup analysis indicates that while neostigmine reduces the overall incidence of PND, its impact on the incidence of POD remains unclear. Consequently, rigorously designed large-sample, multicenter studies are needed to further clarify the role of neostigmine in PND prevention.

Current research indicates that the pathogenesis of PND may involve multiple factors, including central cholinergic system dysfunction (Adam et al., 2020; Downes and Granato, 2004; Cerejeira et al., 2011; John et al., 2017; Cerejeira et al., 2012), abnormal stress responses (Swarbrick and Partridge, 2022), and systemic inflammatory responses induced by surgical trauma and anesthesia (Cheng et al., 2022), among others. Risk factors for PND include the type of surgery, age, perioperative medication and management, pain, use of anticholinergic drugs, frailty, peripheral inflammatory response, pre-existing cognitive impairment (Swarbrick and Partridge, 2022; Cheng et al., 2022; Silbert et al., 2015; Kang et al., 2019; Li et al., 2021), etc. PND is associated with increased postoperative complications, higher mortality rates, reduced quality of life, prolonged hospital stays, and increased healthcare costs (Inouye et al., 2014; Pandharipande et al., 2017; Wilson et al., 2020). Therefore, implementing effective preventive and therapeutic strategies for PND is essential appropriate perioperative interventions serve as one of the primary means for preventing PND, helping to reduce the incidence of postoperative neurocognitive dysfunction (Liu et al., 2022). Previous studies have indicated that some cholinesterase inhibitors may prevent PND (Zhu et al., 2021; Spies et al., 2021) Although it was once believed that neostigmine could not easily cross the blood–brain barrier, recent research suggests that perioperative inflammatory responses and stress might facilitate its passage (Saxena and Maze, 2018; Taylor et al., 2022). Consequently, neostigmine may reduce the incidence of PND by modulating central cholinergic system function (Adam et al., 2020; Downes and Granato, 2004; Cerejeira et al., 2011; John et al., 2017; Cerejeira et al., 2012; Cheng et al., 2022) and mitigating inflammatory responses and oxidative stress (Deng et al., 2019; Zhang et al., 2019; Umholtz and Nader, 2017).

Research on the effects of neostigmine on PONV has produced conflicting results (Ding et al., 1994; Hovorka et al., 1997). In our study, we conducted a meta-analysis of seven studies Deng et al. (2024), Purohit et al. (2022), Zhu et al. (2020), Shuai and Guan Yun (2015), Brueckmann et al. (2015), Hang et al. (2023), and Zhu (2017) to evaluate the impact of neostigmine on PONV incidence (log(OR): 0.25, 95% CI [−0.03, 0.54]; OR: 1.28, 95% CI: [0.97, 1.72], *p* = 0.08, *I*^2^ = 0.00%). Although the neostigmine group exhibited a higher incidence of PONV compared to the control group, the difference was not statistically significant. A previous meta-analysis by Cheng et al. (2005), which included 10 studies, similarly found that neostigmine did not increase the incidence of PONV. Given the variability in control group treatments, perioperative medication use, and types of surgery across the included studies, these results should be interpreted with caution.

When interpreting the results of this study, several limitations should be considered. Firstly, the included clinical studies vary in the types and diagnostic standards of PND, utilizing different neurocognitive assessment tools, such as MMSE, CAM, MoCA, and MDAS, with two studies not specifying their diagnostic methods. This inconsistency in assessment methods introduces considerable variability, affecting the reliability and comparability of the results. The lack of standardized diagnostic criteria highlights the need for caution in interpreting these findings, as differences in diagnostic tools could potentially impact the overall conclusions. Secondly, the types of surgeries and perioperative management and medication varied among the studies, which could affect the incidence of PND and introduce potential bias. Additionally, the control group treatments differed, including the use of Sugammadex and saline, with three studies not clearly describing the control measures, potentially confounding the results. Furthermore, the use of varying dosages of neostigmine across the included studies, combined with the heterogeneity in study design (including both RCTs and cohort studies), may have reduced the accuracy and statistical power of the analysis, potentially influencing the evaluation of neostigmine’s effect on PND. Therefore, these results should be interpreted with caution.

In summary, neostigmine shows a positive effect in reducing the incidence of PND. While it significantly lowers the occurrence of dNCR, its impact on POD remains uncertain. Given the limitations of this study, further large-scale and rigorously designed studies are required to more fully evaluate the potential role of neostigmine in PND prevention.

## Data Availability

The original contributions presented in the study are included in the article/Supplementary material, further inquiries can be directed to the corresponding author.
