# Supplementary material for: Effects of neostigmine on postoperative neurocognitive dysfunction: a systematic review and meta-analysis
Source: Front Neurosci. 2025 Mar 7;19:1464272. doi: 10.3389/fnins.2025.1464272 (PMC11925933; doi:10.3389/fnins.2025.1464272)
Supplement: Supplementary file 1 [file Table_1.DOCX]

| Database | Search terms | Results |
| --- | --- | --- |
| Pubmed | (((((((((((((((((("Neurocognitive Disorders"[Mesh]) OR (NCD[Title/Abstract])) OR (PND[Title/Abstract])) OR (neurocognitive disorder[Title/Abstract])) OR (perioperative neurocognitive disorders[Title/Abstract])) OR ("Emergence Delirium"[Mesh])) OR ("Delirium "[MESH])) OR ("Postoperative Complications "[MESH])) OR (POD[Title/Abstract])) OR (Delirium[Title/Abstract])) OR (Emergence Delirium[Title/Abstract])) OR (postoperative delirium[Title/Abstract])) OR (delayed neurocognitive recovery[Title/Abstract])) OR ("Postoperative Cognitive Complications"[Mesh])) OR (POCD[Title/Abstract])) OR (postoperative cognitive disorder[Title/Abstract])) OR (postoperative cognitive[Title/Abstract])) OR (postoperative cognitive dysfunction[Title/Abstract])) AND (("Neostigmine"[Mesh]) OR ((((((((Synstigmin[Title/Abstract]) OR (Proserine[Title/Abstract])) OR (Prozerin[Title/Abstract])) OR (Neostigmine Bromide[Title/Abstract])) OR (Bromide, Neostigmine[Title/Abstract])) OR (Syntostigmine[Title/Abstract])) OR (Neostigmine Methylsulfate[Title/Abstract])) OR (Methylsulfate, Neostigmine[Title/Abstract]))) | 125 |
| Cochrane | #1 MeSH descriptor: [Neurocognitive Disorders] explode all trees 17824  #2 MeSH descriptor: [Neurocognitive Disorders] explode all trees 17824  #3 MeSH descriptor: [Neurocognitive Disorders] explode all trees 17824  #4 MeSH descriptor: [Neurocognitive Disorders] explode all trees 17824  #5 (NCD):ti,ab,kw OR (PND):ti,ab,kw OR (neurocognitive disorder):ti,ab,kw OR (perioperative neurocognitive disorder):ti,ab,kw OR (POD):ti,ab,kw (Word variations have been searched) 5671  #6 (Delirium):ti,ab,kw OR (Emergence Delirium):ti,ab,kw OR (postoperative delirium):ti,ab,kw OR (delayed neurocognitive recovery):ti,ab,kw OR (POCD):ti,ab,kw (Word variations have been searched) 6329  #7 (postoperative cognitive):ti,ab,kw OR (postoperative cognitive disorder):ti,ab,kw OR (postoperative cognitive dysfunction):ti,ab,kw (Word variations have been searched) 3577  #8 #1or#2or#3or#4or#5or#6or#7 28844  #9 (Neostigmine):ti,ab,kw OR (Synstigmin):ti,ab,kw OR (Proserine):ti,ab,kw OR (Prozerin):ti,ab,kw OR (Polstigmine):ti,ab,kw (Word variations have been searched) 1754  #10 MeSH descriptor: [Neostigmine] explode all trees 623  #11 (Neostigmine Bromide):ti,ab,kw OR (Bromide, Neostigmine):ti,ab,kw OR (Syntostigmine):ti,ab,kw OR (Neostigmine Methylsulfate):ti,ab,kw OR (Methylsulfate, Neostigmine):ti,ab,kw (Word variations have been searched) 230  #12 #9or#10or#11 1754  #13 #8and#12 in Trials 69 | 69 |
| Embase | (('postoperative cognitive dysfunction'/exp OR  'delirium'/exp OR 'emergence agitation'/exp OR  'disorders of higher cerebral function'/exp) OR  'neurocognitive disorder':ab,kw,ti OR  'perioperative neurocognitive disorder':ab,kw,ti  OR 'pod':ab,kw,ti OR 'delirium':ab,kw,ti OR  'postoperative delirium':ab,kw,ti OR 'delayed  neurocognitive recovery':ab,kw,ti OR  'pocd':ab,kw,ti OR 'postoperative  cognitive':ab,kw,ti OR 'postoperative cognitive  disorder':ab,kw,ti OR 'postoperative cognitive  dysfunction':ab,kw,ti) AND ('neostigmine'/exp OR  'neostigmine':ab,kw,ti OR 'synstigmin':ab,kw,ti  OR 'proserine':ab,kw,ti OR 'prozerin':ab,kw,ti OR  'polstigmine':ab,kw,ti OR 'neostigmine  bromide':ab,kw,ti OR 'bromide,  neostigmine':ab,kw,ti OR 'syntostigmine':ab,kw,ti  OR 'neostigmine methylsulfate':ab,kw,ti OR  'methylsulfate, neostigmine':ab,kw,ti) AND  [humans]/lim AND [clinical study]/lim | 377 |
| Scopus | ( ( TITLE-ABS-KEY ( "Neurocognitive Disorders" ) OR TITLE-ABS-KEY ( "NCD " ) OR TITLE-ABS-KEY ( "PND" ) OR TITLE-ABS-KEY ( "neurocognitive disorder " ) OR TITLE-ABS-KEY ( "perioperative neurocognitive disorder" ) OR TITLE-ABS-KEY ( "Emergence Delirium" ) OR TITLE-ABS-KEY ( "Delirium" ) OR TITLE-ABS-KEY ( "POD" ) OR TITLE-ABS-KEY ( "postoperative delirium" ) OR TITLE-ABS-KEY ( "delayed neurocognitive recovery" ) OR TITLE-ABS-KEY ( "Postoperative Cognitive Complications" ) OR TITLE-ABS-KEY ( "POCD" ) OR TITLE-ABS-KEY ( "postoperative cognitive" ) OR TITLE-ABS-KEY ( "postoperative cognitive disorder" ) OR TITLE-ABS-KEY ( "postoperative cognitive dysfunction" ) ) ) AND ( ( TITLE-ABS-KEY ( "Neostigmine" ) OR TITLE-ABS-KEY ( "Synstigmin" ) OR TITLE-ABS-KEY ( "Proserine" ) OR TITLE-ABS-KEY ( "Prozerin" ) OR TITLE-ABS-KEY ( "Polstigmine" ) OR TITLE-ABS-KEY ( "Neostigmine Bromide" ) OR TITLE-ABS-KEY ( "Bromide, Neostigmine" ) OR TITLE-ABS-KEY ( "Syntostigmine" ) OR TITLE-ABS-KEY ( "Neostigmine Methylsulfate" ) OR TITLE-ABS-KEY ( "Methylsulfate, Neostigmine" ) ) ) AND ( LIMIT-TO ( DOCTYPE , "ar" ) ) | 158 |
| Sinomed | (("谵妄"[不加权:扩展] OR "苏醒谵妄"[不加权:扩展] OR "苏醒谵妄"[不加权:扩展] OR "神经认知障碍"[不加权:扩展]) OR "术后认知并发症"[不加权:扩展]) OR "神经认知障碍"[不加权:扩展] OR "认知功能障碍"[不加权:扩展]  2) "新斯的明"[不加权:扩展] OR "neostigmine "[不加权:扩展]  3) ("新斯的明"[不加权:扩展] OR "neostigmine "[不加权:扩展]) AND ((("谵妄"[不加权:扩展] OR "苏醒谵妄"[不加权:扩展] OR "苏醒谵妄"[不加权:扩展] OR "神经认知障碍"[不加权:扩展]) OR "术后认知并发症"[不加权:扩展]) OR "神经认知障碍"[不加权:扩展] OR "认知功能障碍"[不加权:扩展]) | 9 |
| CNKI | (主题：围术期神经认知障碍)OR(主题：谵妄)OR(主题：认知功能) OR (主题：pod)OR(主题：pocd)AND(主题：新斯的明)OR(主题：neostigmine) | 10 |
| Web of Science | 1: (((((((((((((((TS=(Neurocognitive Disorders)) OR TS=(NCD)) OR TS=(PND )) OR TS=(neurocognitive disorder )) OR TS=(perioperative neurocognitive disorder)) OR TS=(Emergence Delirium)) OR TS=(Delirium)) OR TS=(Postoperative Complications)) OR TS=(POD)) OR TS=(postoperative delirium)) OR TS=(delayed neurocognitive recovery)) OR TS=(Postoperative Cognitive Complications)) OR TS=(POCD)) OR TS=(postoperative cognitive)) OR TS=(postoperative cognitive disorder)) OR TS=(postoperative cognitive dysfunction)  2: (((((((((TS=(Neostigmine)) OR TS=(Synstigmin)) OR TS=(Proserine)) OR TS=(Prozerin)) OR TS=(Polstigmine)) OR TS=(Neostigmine Bromide)) OR TS=(Bromide, Neostigmine)) OR TS=(Syntostigmine)) OR TS=(Neostigmine Methylsulfate)) OR TS=(Methylsulfate, Neostigmine)  3: #2 AND #1  4: #3 and Clinical Trial (Document Types) | 56 |
