# Supplementary material for: Effects of neostigmine on postoperative neurocognitive dysfunction: a systematic review and meta-analysis
Source: Front Neurosci. 2025 Mar 7;19:1464272. doi: 10.3389/fnins.2025.1464272 (PMC11925933; doi:10.3389/fnins.2025.1464272)
Supplement: Supplementary file 2 [file Table_2.DOCX]

**Appendix 5: The Egger's test for the effect of Neostigmine on PND after sensitivity analysis.**

Egger's test

------------------------------------------------------------------------------

Std_Eff | Coefficient Std. err. t P>|t| [95% conf. interval]

-------------+----------------------------------------------------------------

slope | -.2065015 .1837206 -1.12 0.294 -.630162 .217159

bias | -.2759698 .6128199 -0.45 0.664 -1.689135 1.137195

------------------------------------------------------------------------------
