# Supplementary material for: Effects of neostigmine on postoperative neurocognitive dysfunction: a systematic review and meta-analysis
Source: Front Neurosci. 2025 Mar 7;19:1464272. doi: 10.3389/fnins.2025.1464272 (PMC11925933; doi:10.3389/fnins.2025.1464272)
Supplement: Supplementary file 3 [file Table_3.DOCX]

**Appendix 9: The Egger's test of effects of POVN.**

Egger's test

------------------------------------------------------------------------------

Std_Eff | Coefficient Std. err. t P>|t| [95% conf. interval]

-------------+----------------------------------------------------------------

slope | .1932657 .2321551 0.83 0.443 -.4035081 .7900394

bias | .189731 .6096448 0.31 0.768 -1.377411 1.756873
