# Supplementary figures and images for: Effects of neostigmine on postoperative neurocognitive dysfunction: a systematic review and meta-analysis
Source: Front Neurosci. 2025 Mar 7;19:1464272. doi: 10.3389/fnins.2025.1464272 (PMC11925933; doi:10.3389/fnins.2025.1464272)

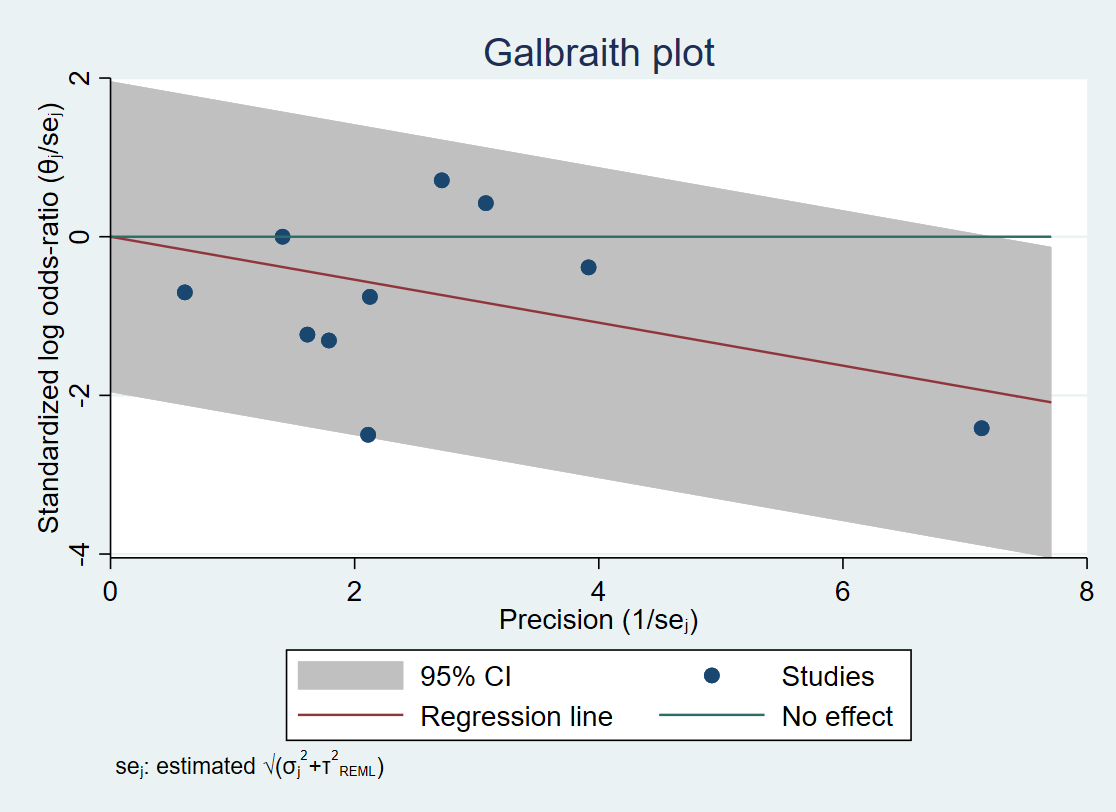

Supplement: Supplementary file 4 [file Image_1.TIF]

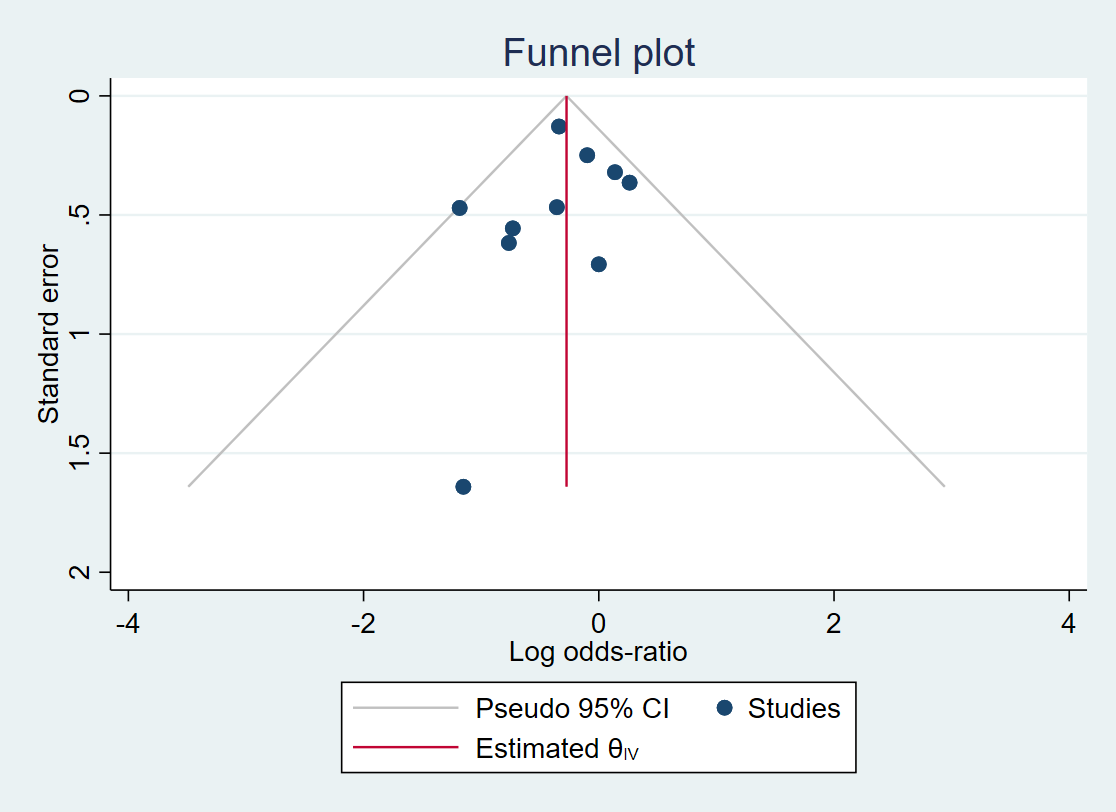

Supplement: Supplementary file 5 [file Image_2.TIF]

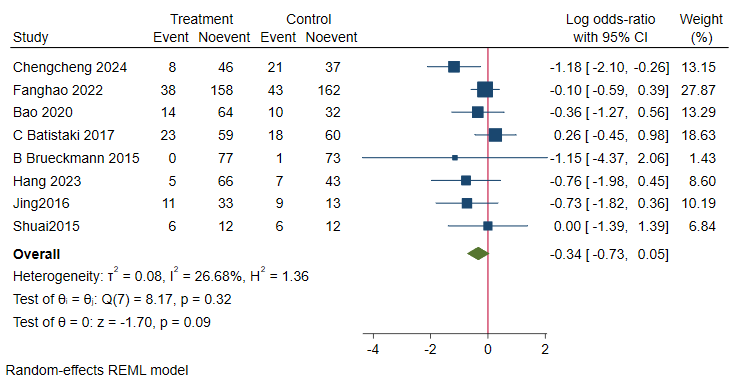

Supplement: Supplementary file 6 [file Image_3.TIF]

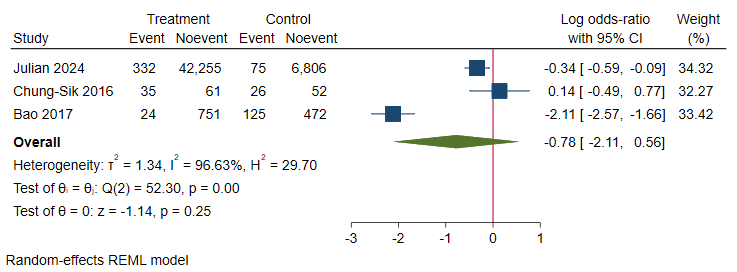

Supplement: Supplementary file 7 [file Image_4.TIF]

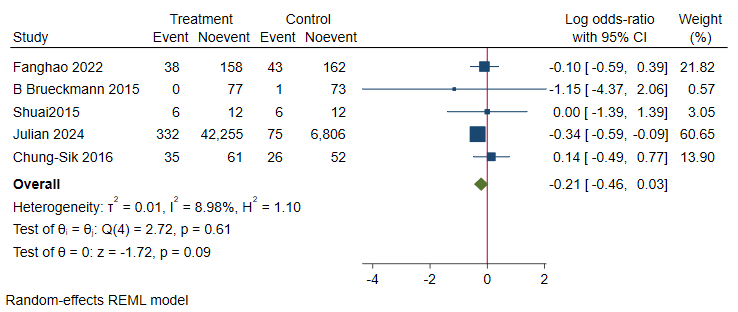

Supplement: Supplementary file 8 [file Image_5.TIF]

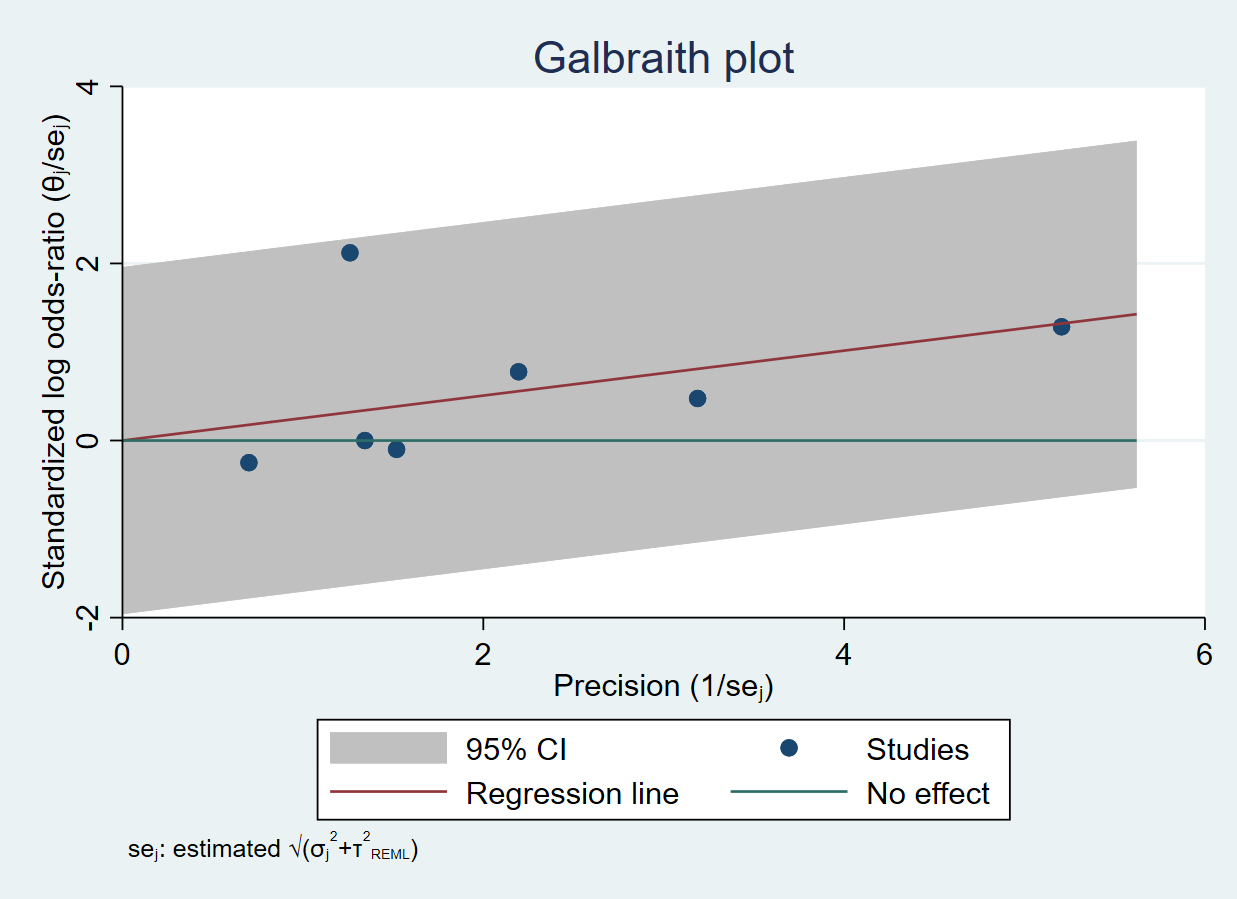

Supplement: Supplementary file 9 [file Image_6.TIF]

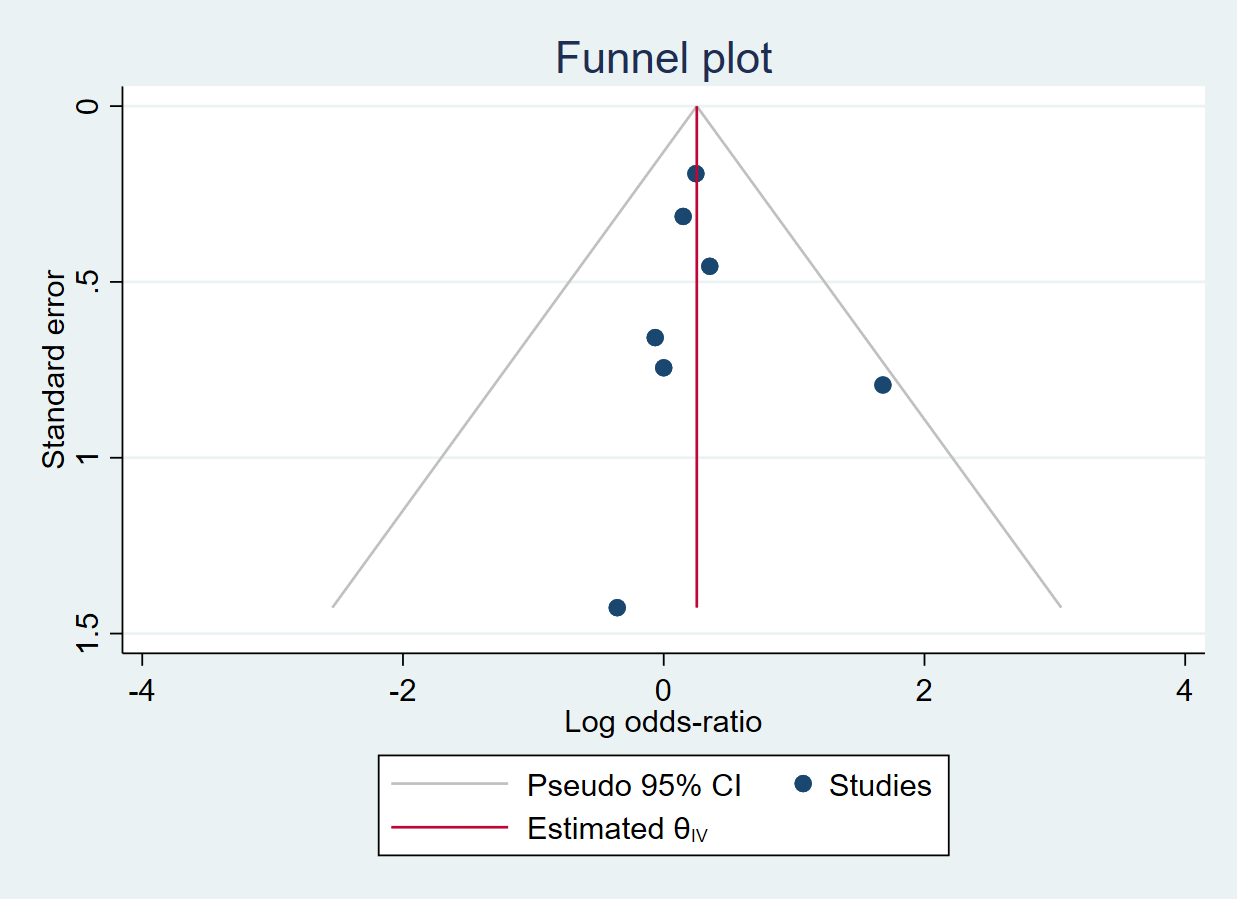

Supplement: Supplementary file 10 [file Image_7.TIF]
